# Abundance, distribution and diversity of gelatinous predators along the northern Mid-Atlantic Ridge: A comparison of different sampling methodologies

**DOI:** 10.1371/journal.pone.0187491

**Published:** 2017-11-02

**Authors:** Aino Hosia, Tone Falkenhaug, Emily J. Baxter, Francesc Pagès

**Affiliations:** 1 Department of Natural History, University Museum of Bergen, University of Bergen, Bergen, Norway; 2 Institute of Marine Research, Flødevigen, Norway; 3 North West Wildlife Trusts, Plumgarths, Kendal, Cumbria, England; 4 Institut de Ciencies del Mar (CSIC), Barcelona, Spain; Evergreen State College, UNITED STATES

## Abstract

The diversity and distribution of gelatinous zooplankton were investigated along the northern Mid-Atlantic Ridge (MAR) from June to August 2004.Here, we present results from macrozooplankton trawl sampling, as well as comparisons made between five different methodologies that were employed during the MAR-ECO survey. In total, 16 species of hydromedusae, 31 species of siphonophores and four species of scyphozoans were identified to species level from macrozooplankton trawl samples. Additional taxa were identified to higher taxonomic levels and a single ctenophore genus was observed. Samples were collected at 17 stations along the MAR between the Azores and Iceland. A divergence in the species assemblages was observed at the southern limit of the Subpolar Frontal Zone. The catch composition of gelatinous zooplankton is compared between different sampling methodologies including: a macrozooplankton trawl; a Multinet; a ringnet attached to bottom trawl; and optical platforms (Underwater Video Profiler (UVP) & Remotely Operated Vehicle (ROV)). Different sampling methodologies are shown to exhibit selectivity towards different groups of gelatinous zooplankton. Only ~21% of taxa caught during the survey were caught by both the macrozooplankton trawl and the Multinet when deployed at the same station. The estimates of gelatinous zooplankton abundance calculated using these two gear types also varied widely (1.4 ± 0.9 individuals 1000 m^-3^ estimated by the macrozooplankton trawl vs. 468.3 ± 315.4 individuals 1000 m^-3^ estimated by the Multinet (mean ± s.d.) when used at the same stations (n = 6). While it appears that traditional net sampling can generate useful data on pelagic cnidarians, comparisons with results from the optical platforms suggest that ctenophore diversity and abundance are consistently underestimated, particularly when net sampling is conducted in combination with formalin fixation. The results emphasise the importance of considering sampling methodology both when planning surveys, as well as when interpreting existing data.

## Introduction

Cnidarians and ctenophores are an important group of pelagic predators. Nevertheless, zooplankton surveys often primarily target crustacean zooplankton, neglecting the diversity and abundance of gelatinous species. This may be due to established sampling protocols that exclude gelatinous zooplankton, a lack of taxonomic expertise, or a belief that standard zooplankton collection methodologies do not provide usable material for the more fragile gelatinous fauna. While the latter indeed appears to be the case for ctenophores, which often require specialised sampling protocols due to their fragility and difficulties with preservation [1]; it is not necessarily true for pelagic cnidarians. Useful data on the diversity and distribution of pelagic cnidarians can be gained from regular zooplankton or micronekton sampling where gelatinous zooplankton is not specifically being targeting (e.g. [2, 3]. Such observations are particularly interesting as information on gelatinous zooplankton assemblages and abundances are historically scarce compared to those of crustacean zooplankton [4, 5]. These data are also crucial for understanding how ongoing anthropogenically-induced changes are having an impact on the faunal assemblages and distribution of gelatinous zooplankton.

Pelagic ctenophores and cnidarians range in size from tiny cydippid ctenophores and hydromedusae that are just a few millimetres in length to large cestid and lobate ctenophores, scyphozoan jellyfish, or colonial siphonophores that can reach several meters in length. Many gelatinous species occur in patches or in relatively low abundances. As such, it is necessary to use range of different gear types and large filtered volumes to target the various components of the gelatinous fauna, or to get a comprehensive overview of the species composition found at any given location and time. However, most studies only use a single sampling method, or a few at most, which has implications for the range of taxa caught. Few studies have compared the selectivity and efficacy of different methodologies in studying gelatinous fauna (but see [6, 7]). Sampling conducted during the RV G.O. Sars MAR-ECO survey along the northern Mid-Atlantic Ridge (MAR) in 2004 [8] offers an opportunity for such comparison. Various net-based and optical methods were used [9] allowing the comparison of the abundance, diversity and distribution of pelagic cnidarians and ctenophores sampled using different techniques.

Here, previously unpublished data on the diversity and distribution of pelagic cnidarians along the northern MAR, sampled with macrozooplankton trawl [9], are presented. Furthermore, the current results are compared with previously published data on pelagic cnidarians and ctenophores from the same survey. Comparisons are based on material collected with the smaller Multinet and a ring net attached to a trawl [2], as well as with observations from optical platforms [10, 11]. In addition to increasing our understanding of the cnidarian diversity along the northern MAR, the present study highlights that the gear type selected for future surveys should be carefully considered, and data from existing studies shout be cautiously interpreted in light of the potential for highly variable results.

## Material and methods

All sampling and field work was carried out in international waters. The shiptime on RV G.O. Sars was approved by the Norwegian Ministry of Fisheries. No protected species were sampled, and no sampling was performed in marine protected areas.

### Study area and survey

**Fig 1:**
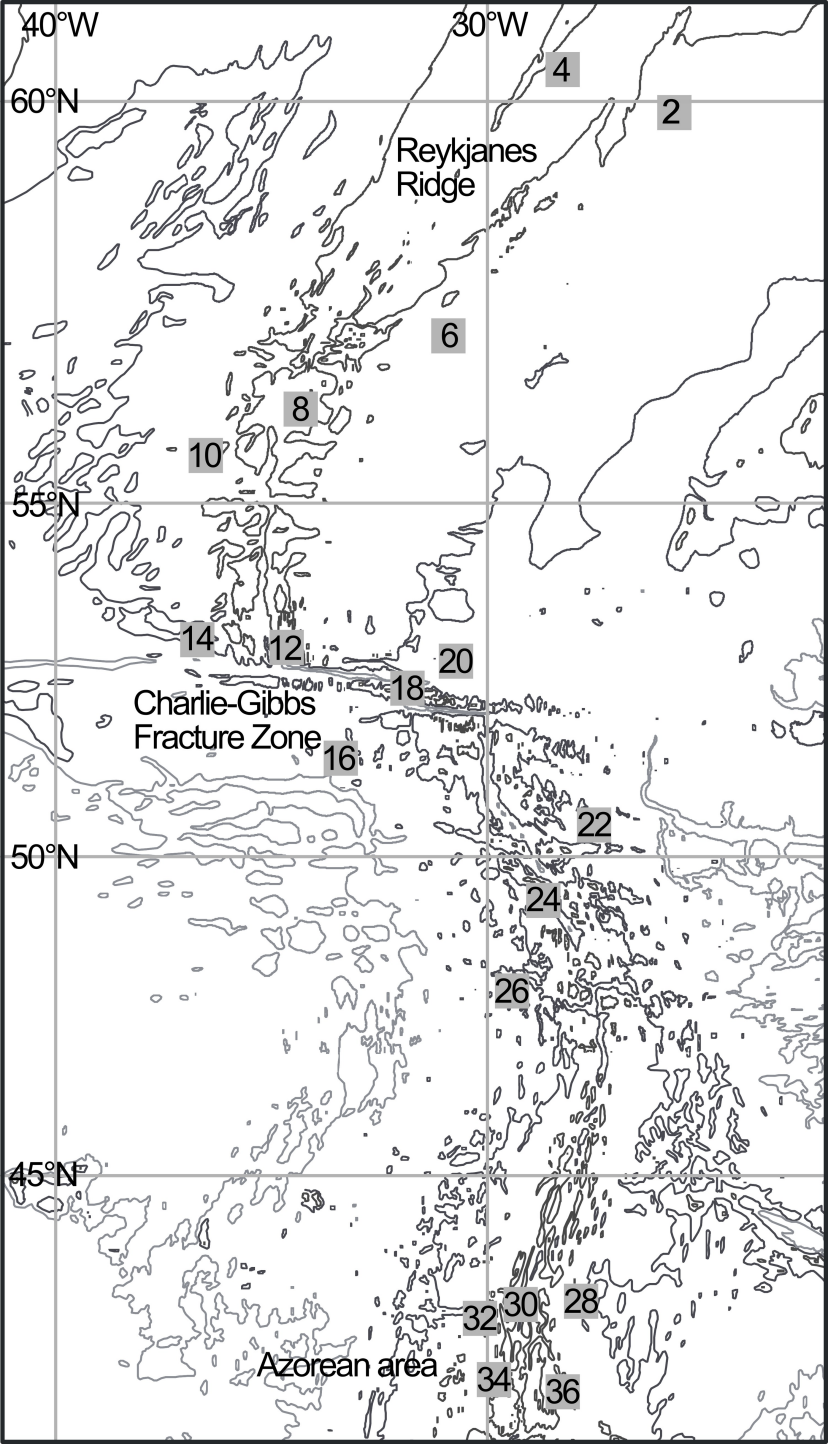
Map of the studied portion of the Mid-Atlantic Ridge. Numbers indicate the locations of the superstations sampled with the macrozooplankton trawl during Leg 1 of the cruise (no data from SS26 due to gear malfunction). Sampling during the return Leg 2 was concentrated around the Azores area and the CGFZ. For exact locations refer to Wenneck *et al*. [9].

Sampling was carried out on board the RV G.O. Sars as part of the MAR-ECO expedition along the MAR between 5 June and 7 August 2004. The pre-defined cruise track included several cross-ridge sections along the northern MAR from Iceland (~60°N—26°W) to the Azores (~41°N—28°W) (Leg 1) and back (Leg 2) (Fig 1). Sampling included several gear types in an effort to sample a wide range of organism sizes and taxa from the surface down to >3000 m.

The MAR is characterised by a complicated topography with depths ranging from 4500 m in the deepest channel to 700–800 m on adjacent seamounts. The Reykjanes Ridge stretches southeast from Iceland and is separated from the southern survey area (the Azores) by the 4500 m deep CGFZ. The topography of the MAR has a profound impact on the circulation pattern in the North Atlantic [12], which in turn is likely to affect the distribution of pelagic organisms. A major hydrographic feature in this region is the Subpolar Frontal Zone (SPF), which crosses the ridge at the CGFZ and creates the division between the North Atlantic Drift Province and the Atlantic Subarctic Province [13]. The MAR-ECO survey covered both provinces with the SPF crossing the middle of the study area.

A detailed description of the physical-oceanographic conditions during the G.O. Sars cruise and the characteristics of the water masses in the area are provided by Søiland *et al*. [14]. The study area was divided into three main regions based on the dominant water masses: Modified North Atlantic Water (MNAW, 6.6°C<*θ*<9°C, sigma−*θ*∼27.4 [14]) dominated the area north of 57°N on the Reykjanes Ridge (superstations (SS) 2 & 4); Sub-Arctic Intermediate Water (SAIW, 5°C<*θ*<9°C, *S*<35 [14]) was found immediately north of the CGFZ and between 52°N and 57°N (SS6-14); and warm and saline North Atlantic Central Water (NACW, *θ*>7°C, *S*>35 [14]) dominated the area south of 48°N (SS28-36). The SPF separating the MNAW and the SAIW was, at the time of our investigation, located at the southern end of the CGFZ, at 52°N 30°W. However, the SPF was not a distinct front. The area between SAIW and NACW formed a broad frontal region (48°-52°N) that was influenced by both water masses (SS16-26). In deeper layers, low-saline Labrador Sea Water (LSW, characterised by a salinity minimum at ~1500 m [14]) was observed in the northern part of the cruise track (60°-48°N) at ~1500 m depth [14]. High-saline Mediterranean Water (MW, characterised by a salinity maximum at ~1000 m [14]) was found south of the SPF at intermediate depths (1000–1500 m) at stations east of the ridge (e.g. SS30) [14].

### Sampling and sample processing

The diversity, distribution and abundance of gelatinous zooplankton were obtained using pelagic trawls, a Multinet, and an Underwater Video Profiler (UVP) during Leg 1 of the survey (5 June-3 July 2004). Ring-net sampling, UVP and Remote Operated Vehicle (ROV) observations were conducted during Leg 2 (4 July-7 August 2004).

Sampling with macrozooplankton trawl (or krill trawl) was conducted at 17 predefined superstations (SS2-SS36, excluding SS26) during Leg 1 of the survey (Fig 1, Table 1). The macrozooplankton trawl is a double warp mid-water trawl with standard, pelagic-trawl doors, a 6 x 6 m mouth opening, 3 x 3 mm mesh (6 mm, stretched), and a total length of 45 m from the mouth to the cod end [9]. The trawl was equipped with a multiple opening–closing device (MultiSampler) [15] and five cod-ends to enable depth-stratified sampling (S1 Table). Each cod-end was equipped with a 7 L collection bucket in order to reduce damage to the animals sampled. The trawl was towed along an oblique trajectory from a maximum depth of 3000 m to the surface (towing speed 2 knots, hauling speed 25 m·min^-1^). The volume of water filtered was calculated for each cod-end by assuming movement at constant speed along an oblique trajectory and a constant mouth opening of 36 m^2^ [9], with the resulting calculated volume of water filtered per tow being 2–4.5x10^5^ m^3^. Data on actual position and depth was provided by SCANMAR sensors attached to the trawl. See Wenneck *et al*. [9] for additional sampling details.

**Table 1:** Missing subsamples from all superstations. Five depth strata were sampled at each superstation. Depth max and depth min indicate the total depth range sampled, while the specific depth strata from which a subsample is missing is provided under comments.

SS	Depth max (m)	Depth min (m)	Comments
2	2141	11	No missing samples
4	1329	5	164–5 m: 1 jar missing (*Periphylla*) 1302–744 m: 3 jars missing (*Periphylla*, *Atolla* & minor subsample)
6	2155	2	No missing samples
8	1337	0	1244–762 m: 1 Cnidaria sample (67 g) discarded on-board. 1337–1328 m: 1 out of 2 subsamples frozen.
10	1986	7	1480–744 m: Lost cod end: No sample
12	1532	7	No missing samples
14	2534	25	No missing samples
16	3008	36	1488–674 m: 1 jar missing (*Atolla*)
18	2660	11	2317–1444 m: 1 jar missing 2660–2320 m: 1 jar missing
20	2527	2	202–2 m: 1 jar missing 676–187 m: 1 jar missing
22	2731	36	No missing samples
24	2768	27	211–27 m: 1 jar missing ("Jellies" 380g)
26	All depths		Gear malfunction–no samples
28	2295	7	No missing samples
30	2383	36	186–36 m: 1 Cnidaria sample (45 g) discarded on-board. 2383–2265 m: 1 jar missing
32	2031	1	No missing samples
34	1981	0	No missing samples
36	2042	0	No missing samples

Gelatinous organisms were separated from the catch and large scyphozoans (*Periphylla periphylla* and *Atolla* spp.) were counted, weighed (wet weight) and discarded. Remaining smaller specimens were bulk fixated in 4% borax-buffered formaldehyde in seawater for later identification and enumeration.

Formalin-fixed gelatinous zooplankton samples, primarily planktonic cnidarians, were identified during two intensive workshops at the University Museum of Bergen, from 16–20 October 2006 (participants A. Hosia, T. Falkenhaug and F. Pagès) and 20–25 January 2012 (participants A. Hosia, T. Falkenhaug and E. Baxter). In addition, E. Baxter identified samples taken to ICM-CSIC, Barcelona, by F. Pagès following the 2006 workshop during her stay there in 2011. Unfortunately, a small proportion of sub-samples from the macrozooplankton trawl could not be located or had been accidentally discarded during sampling (Table 1). Many specimens were also in a relatively poor condition rendering identification to species level impossible. For example, the abundant hydromedusae Halicreatidae were only identified to family level (Table 2 & S1 Table). While only presence/absence of siphonophores are presented in Table 2, all identified zooids and stages were counted separately (presented in S1 Table).

**Table 2:** Pelagic cnidarians and ctenophores caught by macrozoozooplankton trawl at the different superstations, sorted according to taxonomic order. *‘*Hydromedusae’ refers to specimens not identified further than subclass Hydroidolina. All depth strata sampled are combined for each superstation. Average abundance, over the entire water column, is presented in individuals 100 000 m^-3^. For siphonophores, and stations with missing subsamples, no abundance estimate is provided; + indicates presence. Frequency of occurrence (FO) shows the percentage of stations (n = 17) at which a species was observed. For complete data, see supplementary S1 Table.

	Superstation	2	4	6	8	10	12	14	16	18	20	22	24	28	30	32	34	36	FO %
**Anthoathecata**	*Bythotiara murrayi*		+																**5.9**
	*Calycopsis bigelowi*											0.3							**5.9**
	*Sibogita geometrica*																0.3		**5.9**
	Pandeidae						0.3												**5.9**
**Leptothecata**	*Chromatonema rubrum*		+	0.9	+	+	3.0	6.2	+	+	+	2.3	+		+			0.6	**76.5**
	*Modeeria rotunda*												+						**5.9**
	Leptomedusae								+					0.3					**11.8**
**Siphonophorae**	*Abyla trigona*																	+	**5.9**
	*Abyla* sp.															+		+	**11.8**
	*Abylopsis tetragona*													+		+			**11.8**
	*Abylopsis* sp.													+					**5.9**
	Abylidae													+			+	+	**17.6**
	*Amphicaryon acaule*													+		+			**11.8**
	*Amphicaryon ernesti*													+					**5.9**
	*Amphicaryon peltifera*												+						**5.9**
	*Amphicaryon* sp.													+					**5.9**
	*Bassia bassensis*													+		+	+		**17.6**
	*Ceratocymba leuckarti*															+			**5.9**
	*Ceratocymba sagittata*													+			+	+	**17.6**
	*Ceratocymba* sp.													+			+		**11.8**
	*Chelophyes appendiculata*													+	+	+	+	+	**29.4**
	*Chuniphyes multidentata*	+	+	+	+	+	+	+	+	+	+	+	+	+	+	+	+	+	**100.0**
	*Chuniphyes* sp.		+	+		+	+	+		+		+	+	+	+				**58.8**
	*Clausophyes galeata*						+			+									**11.8**
	*Clausophyes moserae*								+										**5.9**
	*Clausophyes* sp.											+					+		**11.8**
	*Diphyes dispar*															+		+	**11.8**
	Diphyidae							+	+										**11.8**
	*Eudoxoides spiralis*																	+	**5.9**
	*Hippopodius hippopus*								+					+	+		+	+	**29.4**
	*Lensia conoidea*		+	+															**11.8**
	*Lensia* sp.			+									+	+					**17.6**
	*Maresearsia praeclara*												+	+			+		**17.6**
	*Nectadamas diomedeae*		+														+	+	**17.6**
	*Nectopyramis natans*																+		**5.9**
	*Nectopyramis thetis*		+	+									+	+		+	+	+	**41.2**
	*Nectopyramis* sp.								+										**5.9**
	*Praya dubia*		+																**5.9**
	*Praya* sp.												+						**5.9**
	Prayidae													+			+	+	**17.6**
	*Rosacea* sp.		+						+			+	+	+			+	+	**41.2**
	*Vogtia glabra*		+						+				+	+			+		**29.4**
	*Vogtia pentacantha*		+			+						+		+	+	+	+	+	**47.1**
	*Vogtia serrata*		+	+				+						+			+		**29.4**
	*Vogtia spinosa*		+											+	+		+		**23.5**
	*Vogtia* sp.		+	+										+			+		**23.5**
	Calycophorae							+										+	**11.8**
	*Agalma okeni*																	+	**5.9**
	*Agalma* sp.																+		**5.9**
	*Apolemia uvaria*																+		**5.9**
	*Bargmannia amoena*													+					**5.9**
	*Halistemma striata*															+			**5.9**
	*Halistemma* sp.			+	+									+	+		+	+	**35.3**
	*Physophora hydrostatica*		+							+								+	**17.6**
	*Stephanomia amphytridis*	+		+				+					+		+		+	+	**41.2**
	Physonectae			+	+				+	+		+	+	+			+	+	**52.9**
**Narcomedusae**	*Aegina citrea*																0.3		**5.9**
	*Aeginura grimaldii*	0.7	+	14.8	+	+	93.1	57.6	+	+	+	41.0	+	7.9	+	24.3	18.2	15.0	**100.0**
	*Cunina duplicata*											0.3							**5.9**
	*Solmissus* sp.					+		0.9											**11.8**
	*Solmissus incisa*							0.6	+		+	0.8	+	0.4	+			0.6	**47.1**
**Trachymedusae**	*Aglantha digitale*		+	8.7	+					+	+	22.3	+						**41.2**
	*Colobonema sericeum*	2.9	+	1.9	+	+	3.0	5.0		+	+	5.7	+	1.8	+	3.2	6.7	6.7	**94.1**
	*Crossota alba*												+						**5.9**
	*Crossota rufobrunnea*				+			1.9		+					+				**23.5**
	*Crossota* sp.				+					+									**11.8**
	Halicreatidae		+	12.9	+	+	64.1	164.0	+	+	+	179.9	+	12.0	+	6.5	65.7	3.6	**94.1**
	*Halitrephes maasi*							0.3	+			0.3	+				0.5		**29.4**
	*Pantachogon haeckeli*					+	15.5	1.2		+	+	1.0					0.3		**41.2**
	*Rhopalonema velatum*												+						**5.9**
	Rhopalonematidae										+	0.3							**11.8**
	Trachymedusae			1.2	+		1.3	21.5	+	+	+		+	0.1			0.3		**58.8**
**Div. Hydroidolina**	Hydromedusae			1.8			3.0	3.4		+				0.3			0.3		**35.3**
**Coronatae**	*Periphylla periphylla*	6.9	+	5.7	+	+	3.0	8.7	+	+	+	17.7	+	2.1	+	3.2	3.5	2.8	**100.0**
	*Atolla* spp.		+	7.5	+	+	2.4	6.2	+	+	+	1.8	+	1.7	+	11.4	0.5	1.4	**94.1**
	*Atolla wyvillei*				+										+				**11.8**
	*Atolla vanhoeffeni*				+			0.3						0.1					**17.6**
	*Paraphyllina* sp.			0.3												0.3			**11.8**
	Coronatae									+							0.3	0.3	**17.6**
**Semaeostomeae**	*Pelagia noctiluca*														0.2				**5.9**
**Beroida**	*Beroe* sp.							0.3				1.0	15.0	10.7	7.4	3.0	5.6	9.4	**47.1**
	**Taxon richness (Cnidaria)**	**5**	**21**	**20**	**15**	**11**	**13**	**20**	**17**	**18**	**12**	**19**	**24**	**36**	**18**	**17**	**36**	**28**	

Previously published data from the same cruise were used for the comparison with the Multinet and trawl-attached ring-net [2], as well as observations made via ROV [10] and UVP [11]. The Multinet (HydroBios– 0.25 m^2^ in diameter and 180 μm mesh size) was towed vertically with a hauling speed of 40 m·min^-1^. Additional, non-quantitative data on species presence were obtained from a ring net (1 m in diameter and 750 μm mesh size) attached to the roof of the bottom trawl [2]. The macrozooplankton trawl data from the current study and the Multinet data [2] come from the same superstations sampled during the first leg of the survey and are therefore compared in more detail. Ring net samples were collected during the return leg of the survey.

### Statistical analyses

**Fig 2:**
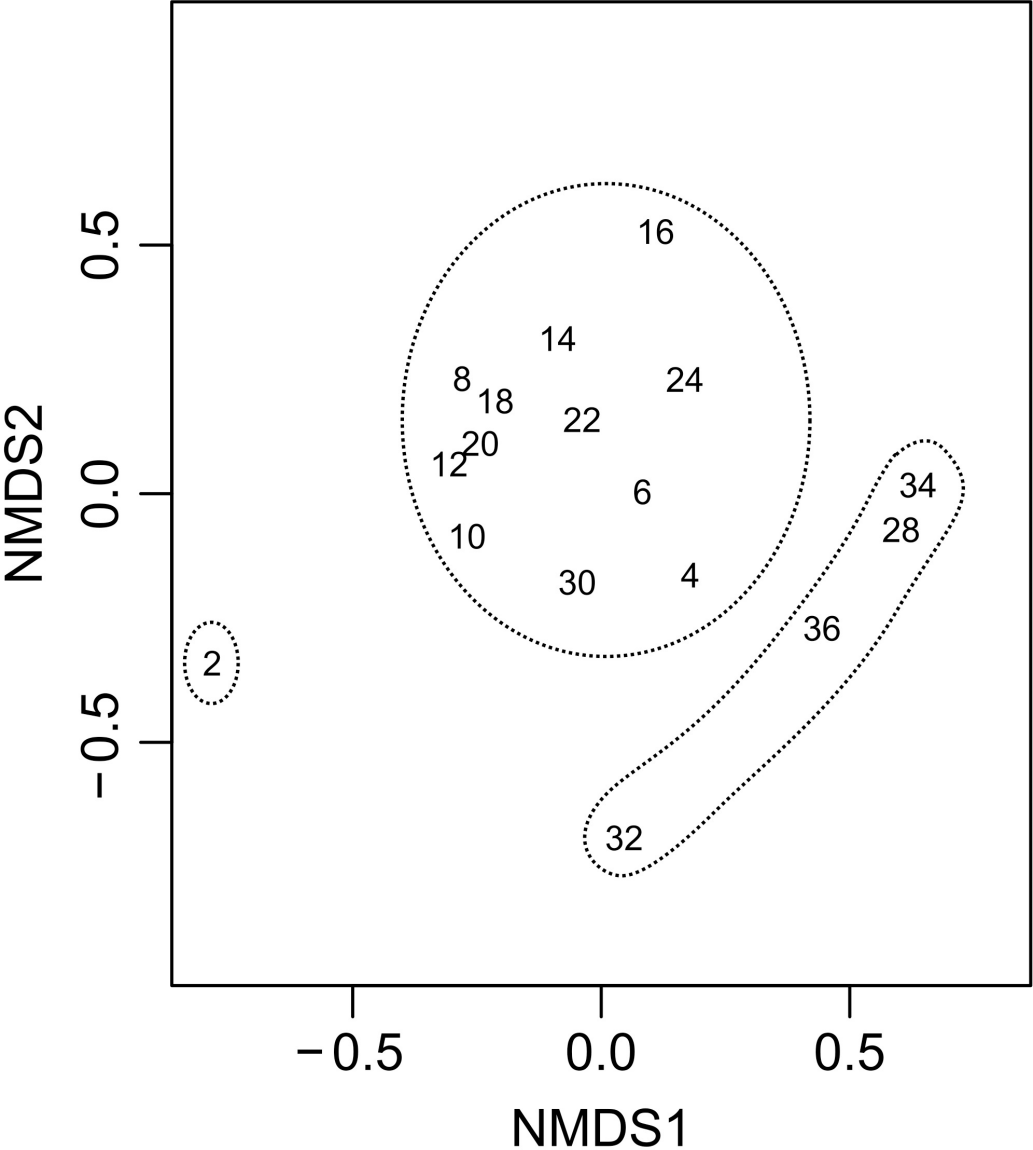
Macrozooplankton trawl samples from different superstations. nMDS (Sørensen distance, stress 0.13) and hierarchical clustering (average linkage) at 60% level of similarity.

Previous studies have suggested that a problem with the closing mechanism on the macrozooplankton trawl might have led to contamination by deep water fauna in samples from shallower strata [16]. As such, samples from the macrozooplankton trawl were pooled for all depths at each station. Due to the uncertainties in enumeration (missing subsamples and uncertain number of siphonophore colonies) and the large proportion of zero values, the Sørensen distance–which treats the data as presence/absence–was used for calculating similarity matrices. Non-metric multidimentional scaling (nMDS) was then used to visualize the data. Hierarchical clustering with average linkage was performed and superimposed on the nMDS (Fig 2). For the statistical comparison of catch composition from the different net based gears (macrozooplankton trawl, Multinet and ring net) the *adonis* function (Sørensen distance, 9999 permutations) was used on presence/absence data of the cnidarian catch. Data from each gear were pooled per station and only macrozooplankton trawl stations without missing subsamples were used for the analysis. As the macrozooplankton trawl and the Multinet were employed at the same stations, catch statistics from these two gears were further compared in more detail. All analyses were done with R version 2.3.2 [17] and the package Vegan [18].

## Results

### Macrozooplankton trawl

A total of 16 species of hydromedusae, 31 species of siphonophores, four species of scyphozoans and one ctenophore species were identified to species level from macrozooplankton trawl samples (Table 2). In addition, a number of specimens were identified to higher taxonomic levels only. While many of these probably represent poorly preserved individuals of the species otherwise observed in the samples, there were also two additional genera only identified to genus level (*Rosacea* sp. and *Paraphyllina* sp.). Only three species, the narcomedusa *Aeginura grimaldii*, the siphonophore *Chuniphyes multidentata*, and the coronate scyphozoan *Periphylla periphylla* were observed at all stations. Other species that were observed at more than half of the stations were the hydromedusae *Chromatonema rubrum* and *Colobonema sericeum*, as well as the *Atolla* spp. scyphozoans.

Hierarchical clustering indicated three clusters at ~60% similarity: SS2 alone, SS4-SS24, and SS28-36 (Fig 2). The major divide between the two larger clusters coincided with the southern limit of the SPF region during the survey. SS2 differed from the other stations due to having much lower species richness. Only five cnidarian taxa were observed at SS2, all of them common throughout the study region. In comparison, the average taxon richness of cnidarians at the rest of the stations was 19.5 (range 10–35, Table 2). However, several hydromedusae, particularly *Aglantha digitale*, were only caught at the northern MNAW, the SAIW-dominated stations, and the SPF region. In contrast, a number of siphonophore species, like the abylids *Chelophyes appendiculata*, *Amphicaryon* spp. and *Diphyes dispar*, were primarily observed at the southern NACW stations (Table 2). While average species richness observed at the stations in the SS28-SS36 cluster (27 ± 9.3, mean ± s.d.) was higher than that in the SS4-SS24 cluster (17.4 ± 4.1), this difference was not statistically significant (Welch’s *t-*test, p = 0.07). A total of 61 cnidarian taxa (range 17–36 taxa per station) were recorded from the five superstations in the SS28-36 cluster, while 55 taxa (range 11–24 taxa per station) were recorded from the 11 superstations in the SS4-SS24 cluster (Table 2).

### Comparisons with other gear

**Fig 3:**
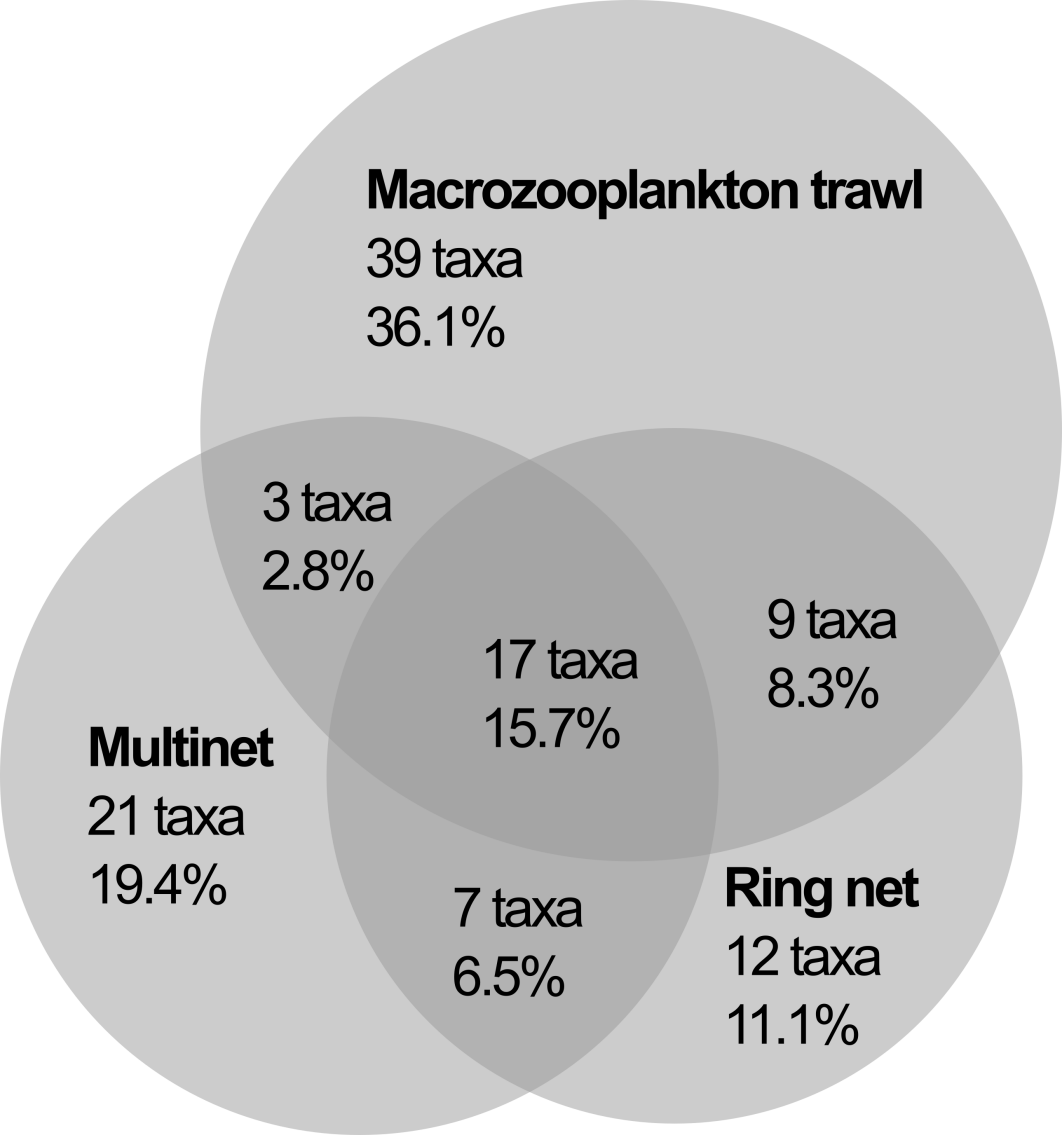
Venn-diagram of pelagic cnidarian taxa (n = 109) caught by different net types during the survey. All identifications to species and/or genus level are included. Halicreatidae in macrozooplankton trawl data are included in the comparison as *Halicreas minimum*, which was positively identified in the samples.

**Fig 4:**
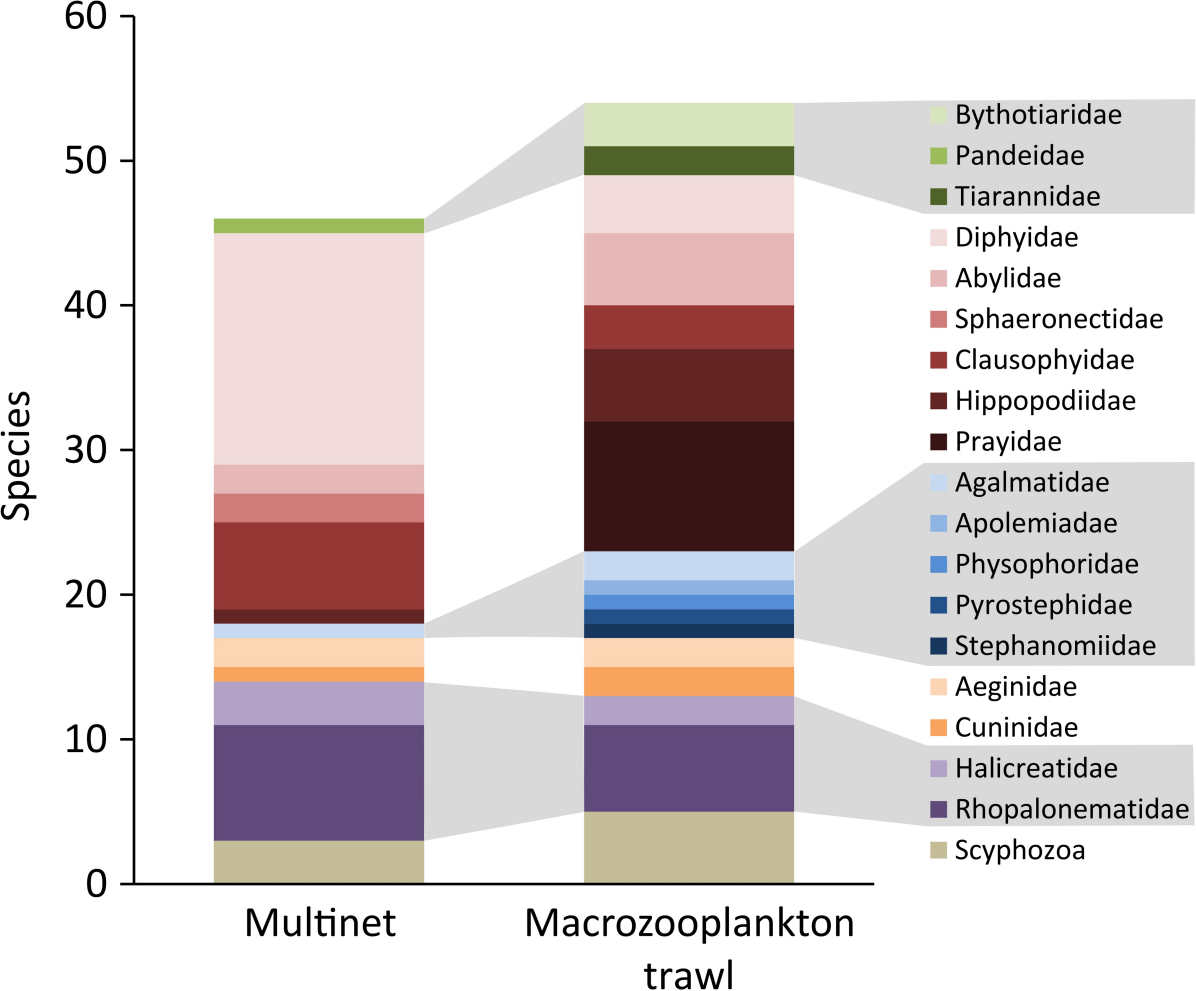
Number of cnidarian species or taxa per family caught by the Multinet and macrozooplankton trawl. The colour coded higher hydrozoan taxa from top to bottom are: Orders Anthoathecata and Leptothecata (green); order Siphonophorae, suborder Calycophorae (red), suborder Physonectae (blue); order Narcomedusae (orange); and order Trachymedusae (purple).

A combined total of 109 species, or higher taxa, of pelagic cnidarians were identified from the three types of net-caught samples (current study, [2]). Less than 16% of the taxa identified were common to all three sampling systems, suggesting a high degree of selectivity by the different gear types (Fig 3). *Adonis* also indicated significant differences between the catch composition from the three different methodologies (p<0.001, R2 = 0.33). A comparison of the catch composition from the macrozooplankton trawl and the Multinet at the stations (Table 3), with no missing subsamples, shows that the two nets did not differ significantly in terms of the mean number of taxa caught per station (Welch’s *t-*test, p = 0.85). However, only on average 13.6% (range 5.9–26.1%) of the taxa sampled were shared between the two net types when used at the same station. A breakdown of the catch into families shows that this selection is not random. The nets appear to target taxonomic groups differently (Fig 4). For example, the Multinet sampled a wider variety of diphyids, while the macrozooplankton trawl was more effective at catching hippopodids, prayids and physonects. In addition to sampling different components of the gelatinous fauna, total abundance estimates from the macrozooplankton trawl are two to four orders of magnitude lower than those from the Multinet (Table 3).

**Table 3:** Comparison between samples from the macrozooplankton trawl and Multinet. Only stations with complete samples (i.e. no missing subsamples) are included. Mean abundance of pelagic cnidarians from the entire sampling volume in individuals 1000 m^-3^. In the taxon count, higher taxa have only been included if the sample contains no species/genera belonging to them.

	Macrozooplankton trawl				Multinet				
SS	Max. depth	Filtered vol. (m^3^)	Average abundance	No. taxa	Max. depth	Filtered vol. (m^3^)	Average abundance	No. taxa	Shared taxa
2	2141	305562	0.11	5	2152	636	1030	17	3
12	1532	296515	1.91	10	1903	573	240	13	3
14	2534	321363	2.86	14	2502	984	170	15	6^a^
28	2294	709933	1.06	24	2507	947	360	12	2^b^
32	2031	378702	0.89	18	1897	624	390	20	4 ^a^
36	2042	360812	1.37	23	1906	582	620	21	3

^a^ Including Halicreatidae in Macrozooplankton trawl vs. any species from the family in Multinet

^b^ Including *Lensia* sp. in Macrozooplankton trawl vs. several species from the genus in Multinet

**Fig 5:**
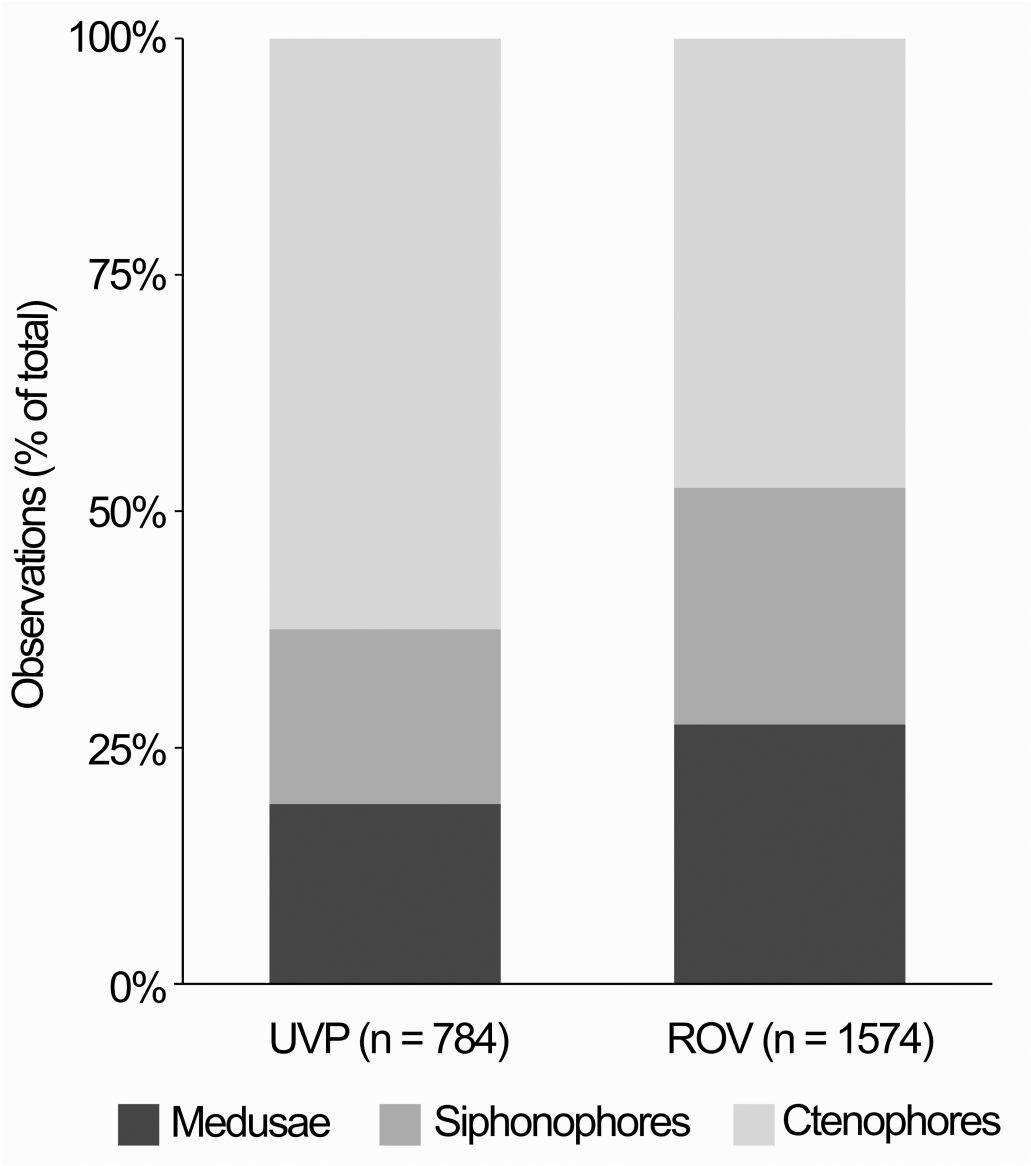
Gelatinous zooplankton observed with the UVP [11] and ROV [10] by group.

Stemman *et al*. [11] recorded a total of 784 observations of ctenophores and cnidarians from UVP deployments made during the MAR-ECO survey, while Youngbluth *et al*. [10] made 1574 observations with the ROV (Fig 5). The observations based on these optical platforms differed from those based on net sampling particularly in terms of number and diversity of ctenophore observations. Only robust beroids were frequently observed in the physical net and trawl samples, accounting for 2.2% of total gelatinous zooplankton specimens in the macrozooplankton trawl (current study) and <1% in the Multinet samples [2]. In contrast, ctenophores contributed 27.4% of the total 1574 observations on ctenophores and pelagic cnidarians made with the ROV [10] and 19.3% of the 784 observations by the UVP [11]. Lobates made up 75–95% of all ctenophore observations with the ROV, with *Bathocyroe fosteri* being the most commonly observed species and *Bolinopsis infundibulum* occurring at 12 out of 14 stations [10]. The rest of the observations were mostly accountable to two species of small unidentified and potentially undescribed mesopelagic cydippids, with only two *Beroe* sp. individuals observed during all dives [10]. Similarly, the UVP images contained 66.9% lobates and 33.1% cydippids, the latter peaking at 400–600 m [11]. The cydippids photographed by the UVP were of two different morphologies. In addition, a single cestid ctenophore was also observed on one of the casts [11].

## Discussion

Biogeographic patterns in the oceans are best known in the epipelagic realm [19, 20]. Information on the deeper pelagic biota is sparser, and the patterns are expected to diverge from those observed at the surface [20]. The SPF is known as the transitional zone between the pelagic provinces of the Subarctic Atlantic in the Northern Coldwater Realm, and the North Atlantic Current in the Atlantic Warm Water Realm [19, 20]. Previous studies have shown that in the study region the SPF acts as a major biogeographic boundary, affecting the species assemblages of pelagic cnidarians [2], mesozooplankton [21, 22], micronekton [22, 23], cephalopods [24] and fish [16], with its effect diminishing with increasing depth [2, 22] and organism size [22]. The analyses presented here, based on the assemblages of pelagic cnidarians caught by the macrozooplankton trawl, follow this pattern with the sampling stations dividing into northern and southern clusters on either side of the southern limit of the SPF. Even though the different depth strata were pooled in our analyses, most of the species we commonly observed throughout the area are considered meso- or bathypelagic. Such species with a high FO in the macrozooplankton trawl catches were *Aeginura grimaldii*, *Chuniphyes multidentata*, *Periphylla periphylla*, *Chromatonema rubrum*, *Colobonema sericeum*, *Atolla* spp. and the Halicreatids [25, 26, 27]. This reflects the previously detected convergence of communities at the deeper depths [2, 22]. Differences in the cnidarian assemblages closer to the surface, previously found to be profoundly different on either side of the SPF [2], probably contributed to the observed clustering in the current study.

While the biogeographic patterns of cnidarian distribution based on the macrozooplankton trawl catches in the current study were largely congruent with the analyses on the catch from the Multinet [2], the two gear types differed considerably in terms of their performance. The average abundance of individuals estimated from the Multinet were consistently at least two orders of magnitude larger than those estimated from the marcozooplankton trawl. This was despite different parts of siphonophores being considered individually from macrozooplankton samples, while attempts were made to estimate the number of colonies with the Multinet. This considerable difference could be due to a couple of different sources. Firstly, the two gears targeted different portions of the fauna. The Multinet, with its smaller mesh size, was more effective at sampling the smaller and conceivably more abundant diphyid siphonophores and small trachymedusae, thus accumulating more individuals. The most abundant species in the Multinet samples were the diphyids *Lensia conoidea*, *Dimophyes arctica*, *Gilia reticulata*, *Eudoxoides spriralis* and *Lensia subtilis*, as well as the trachymedusae *Aglantha digitale* and *Aglaura hemistoma* [2]. These species were either absent or rare (FO 0–11.8%) in the macrozooplankton trawl catch, apart from the relatively frequently occurring *A*. *digitale* (FO 41.2%). The macrozooplankton trawl, with its larger sample volumes, was better at documenting the diversity of large prayid (6 vs. 0 species in the macrozooplankton trawl and the Multinet, respectively), hippopodid (4 vs. 1 species) and physonect (6 vs. 1 species) siphonophores, which are likely to occur at much lower abundances (Fig 4). Secondly, a higher proportion of the gelatinous material may have been lost during the more extensive processing of the macrozooplankton trawl samples prior to fixation. However, this processing bias alone would not be expected to cause such a considerable discrepancy in counts.

Despite its apparently lower catch efficiency, the macrozooplankton trawl contributed significantly to the estimated diversity, being the sole means of sampling more than one third of the species. Again, this is probably largely due to the filtered volume sampled, which was three orders of magnitude larger for the macrozooplankton trawl compared to the Multinet (Table 3). Sampling a larger volume would increase the likelihood of collecting relatively rare species. The macrozooplankton trawl’s contribution to the total number of species sampled could have been even greater, had the samples been in better condition. The poor condition of the samples may have been due to: a) the trawl itself being too rough a method for fragile gelatinous fauna; b) the sorting and processing of the samples prior to fixation; and/or c) poor preservation. The high proportion of Halicreatidae hydromedusae in the macrozooplankton trawl samples can be attributed to their relatively robust mesoglea and distinctive shape, even though the species specific characteristics were often destroyed. Due to the damage sustained, the Halicreatidae were only identified to family level. However, both *Halicreas minimum* and additional species were clearly present in the samples. Given the large number and ubiquity of Halicreatidae caught by the Macrozooplankton trawl compared to the Multinet [2], it seems likely that all the species observed in the Multinet would also be expected to be found in the trawl.

Active avoidance by the jellies was probably of no major importance in causing the observed differences in catch between gears. Skjoldal *et al*. [28] concluded that active avoidance is of small consequence for zooplankton groups other than the fast swimming micronekton such as krill. Heino *et al*. [29] also found that *Atolla* spp. medusae had relatively high catchability compared to a number of fish, cephalopods and decapods, probably due to a combination of large size and slow escape speed.

In their comparison of various net-based zooplankton sampling systems, Skjoldal *et al*. [28] highlighted that in addition to mesh size having an obvious effect on the zooplankton species composition sampled, a high towing speed can substantially increase the extrusion of smaller animals through the net. Such an effect is probably particularly important for soft bodied gelatinous zooplankters. A combination of these factors could have therefore resulted in the low prevalence of smaller gelatinous zooplankters in our macrozooplankton trawl and trawl attached ring-net samples. However, the much larger volumes filtered by the macrozooplankton trawl and the ring-net probably enabled these gears to sample several less abundant species, absent from the Multinet samples. According to Skjoldal *et al*. [28], different net systems produced similar results if they had comparable mesh sizes and sufficiently high mesh open area to mouth opening ratio. This would make the results from–and the limitations of–the type of Multinet we used comparable to those from a standard WP2 with a 0.25 m^2^ opening and 180 μm mesh, widely used for routine plankton surveys. An obliquely-towed, multiple-opening closing device with a 180 μm mesh and relatively large filtered volume, such as MOCNESS [30] or a Multinet with a larger mouth area, could probably have sampled a larger portion of the gelatinous zooplankton diversity alone than any of the methods used in this study. Results from the Euro-Basin survey between Bergen, Norway, and Nuuk, Greenland, suggest that this might be the case [3]. The Euro-Basin survey also utilised several gears at each station to sample zooplankton and micronekton, including a specialised jellynet, a MOCNESS, as well as Harstad and macrozooplankton trawls. Sixteen species or genera were identified from the jellynet (which only sampled the upper 200 m of the water column), 37 from the MOCNESS (0–1000 m) and 32 from the macrozooplankton and Harstad trawls combined, with approximately 70% of the observed species represented in the MOCNESS samples [3]. Jellyfish abundances based on the MOCNESS catch were reported for different depth strata and ranged from 0 to a maximum of 195 individuals 100 m^-3^ in the upper 25 m at one of the stations [3]. However, the average for the entire water column would be much lower than this maximum. Similar to the Multinet in our study, the MOCNESS was able to sample smaller hydrozoans that were typically lacking from the trawl samples [3].

For the larger scyphozoans, jellyfish bycatch from pelagic fish trawling surveys has on several occasions proved useful for estimating their distribution and abundances [31, 32]. In the current study, the macrozooplankton trawl was clearly better than the Multinet for obtaining estimates on the abundance of the common midwater scyphozoans *Periphylla periphylla* and *Atolla* spp. These species were only occasionally collected in the Multinet [2] but were some of the most ubiquitous species in the macrozooplankton trawl samples (Table 2).

The macrozooplankton trawl, Multinet and ring-net together provide data on a wide range of pelagic cnidarians, from tiny hydromedusae and euxodis of diphyid siphonophores, to scyphozoans and large siphonophores. However, large siphonophores were rarely caught and often challenging to identify to species level due to damage sustained during the sampling process. For these groups, the optical methods employed provided limited additional data on diversity, as the image quality often precluded identification to species level. Optical methods were nevertheless vital in providing data on the detailed vertical distribution of the different groups of gelatinous zooplankton, not readily available from the other methodologies [10, 11].

The situation was entirely different for the even more fragile ctenophores. Optical methods provided a fundamentally different–and potentially more realistic–estimate of the relative importance and diversity of ctenophores in the gelatinous community compared to net sampling. Apart from the robust beroids, which appeared more common towards the southern parts of the survey area, no ctenophores were identified from the macrozooplankton trawl samples. This conspicuous absence of ctenophores in the net samples can be attributed to damage suffered by the fragile animals during the sampling process (e.g. *Bathocyroe* spp.) [1, 5] and the subsequent disintegration of preserved specimens in formalin [1, 33, 34]. It may therefore be reasonable to assume that traditional net based methods, particularly if combined with fixation of samples for later analysis, consistently underestimate both the abundance and diversity of ctenophores in the open ocean.

As an alternative to traditional morphological species identification, DNA barcoding is increasingly being used to identify specimens either individually, or in bulk through metabarcoding applications [35, 36, 37]. These approaches hold particular promise for soft bodied animals such as gelatinous zooplankton, where damage incurred during sampling (or ingestion by predators, if stomach contents are the focal interest) may prevent morphological identification. Empirical experience shows that both the proposed universal barcode locus, mitochondrial cytochrome oxidase I (COI), and its common alternative, mitochondrial 16S rRNA, are generally capable of distinguishing between species of pelagic cnidarians [35, 36, 38]. However, their utility for the purposes of identifying individual species is currently limited by the lack of comprehensive and accurate barcode reference databases [35, 36]. For ctenophores, DNA barcoding is still in its infancy, although COI has recently been shown to work for the benthic coeloplanids, and the techniques may thus hold promise for the future [38]. Fixation and preservation protocols pose additional problems for both pelagic cnidarians and ctenophores. For cnidarians, bulk fixation with formalin in sea water allows later morphological identification, but can hinder subsequent DNA sequencing [39]. On the other hand, preservation in ethanol causes distortion and makes the morphological identification of many pelagic cnidarians difficult. Metabarcoding could in the future provide an alternative method to recover diversity information from samples bulk preserved in ethanol, but this requires further development of the methods as well as the reference databases, with the retrieval of quantitative information posing a further challenge [37]. On the other hand, the majority of ctenophores cannot be satisfactorily preserved for later identification and enumeration, and currently require live processing of the samples for estimations of diversity and abundance. Even this will probably only provide data only on the more robust species that can withstand net sampling. Metabarcoding or eDNA methods, when refined, could therefore prove useful for re-evaluating ctenophore diversity and distributions.

## Conclusions

Substantial data on diversity and distribution of pelagic cnidarians could be obtained from net and trawl based plankton sampling conducted during the MAR-ECO survey, even though gelatinous zooplankton were not specifically targeted or accommodated for during the sampling. If samples retrieved using these methods are processed and fixed in a gentle manner, even samples taken during standard plankton surveys can potentially hold large amounts of valuable information on the diversity, distribution and abundance of pelagic cnidarians. The results from the current study obtained using the macrozooplankton trawl considerably increase the number of gelatinous zooplankton species observed during the MAR-ECO survey of the northern Mid-Atlantic Ridge region compared to other net-based methods. They also support earlier conclusions regarding the biogeography of pelagic organisms in the region [22]. However, the comparison of sampling methods utilised during the survey also shows that none of the used gear offers a single catch-all solution to comprehensively sample gelatinous zooplankton diversity. Of particular note is the likely consistent underestimation of diversity and ecological importance of ctenophores based on their considerable underrepresentation in preserved net samples, compared to *in situ* observations. Both the substantial selectivity exhibited by the different gears and the widely diverging density estimates they provided should be considered when planning future surveys, as well as when interpreting existing data on gelatinous zooplankton assemblages and abundances. Important factors to consider are: a suitable mesh size and sufficient filtered volumes with respect to the targeted fauna; a moderate towing speed to limit damage to the animals and extrusion through the net; and gentle processing of retrieved sample to avoid further damage to the collected specimens. Selectivity issues should also be carefully considered when data from different gear types and different surveys are compared or combined for further analysis.

## Supporting information

S1 Table Cnidarians and ctenophores sampled by Macrozooplankton trawl. Abundances per 100 000 m^-3^. Grey columns in bold (SS*i*-full) show the pooled densities calculated over the entire water column for each superstation. Columns in italics indicate samples with missing subsamples (see Table 1). For siphonophores, the type of collected zooids is indicated as follows: an (anterior nectophore), br (bract), col (colony), eud (eudoxid), gon (gonophore), n (nectophore), pc (polygastric colony), pn (pneumatophore).
(PDF)
